# Socioeconomic vulnerability and frailty among community-dwelling older adults: cross-sectional findings from longitudinal aging study in India, 2017–18

**DOI:** 10.1186/s12877-022-02891-1

**Published:** 2022-03-14

**Authors:** Shobhit Srivastava, T. Muhammad

**Affiliations:** International Institute for Population Sciences, Mumbai, Maharashtra 400088 India

**Keywords:** Vulnerability, Frailty, LASI, Older adults, India

## Abstract

**Introduction:**

The Indian population is rapidly aging with huge proportion of illiterate and socioeconomically disadvantaged people and there is a dearth of research on the relationships between factors of socioeconomic vulnerability and frailty in older people. The present study examined the cross-sectional associations between socioeconomic vulnerability and physical frailty in community-dwelling older individuals in India.

**Materials and methods:**

The data for the study were obtained from the Longitudinal Aging Study in India (LASI), which was conducted in 2017–18. The effective sample size was 14,652 older males and 15,899 older females aged 60 and over. The outcome variable was physical frailty phenotype measured from exhaustion, unintentional weight loss, weak grip strength, low physical activity, and slow walking time. The main explanatory variable was vulnerability status based on education, wealth and caste. The study carried out bivariate analysis to observe the association between vulnerability status and physical frailty. Further, multivariable binary logistic regression analysis was conducted to fulfil the objective of the study.

**Results:**

A proportion of 10.5 and 14.4% of older males and females respectively were in the overall vulnerable category. The prevalence of physical frailty was high among older males from vulnerable population (31.4% vs 26.9%; *p* < 0.001). The adjusted estimates from multivariate analysis revealed that older adults from vulnerable category had 14% significantly higher odds of being frail in comparison to non-vulnerable category [AOR: 1.14; CI: 1.06,1.24]. The adjusted model further revealed that there were no significant gender differentials in physical frailty among older adults. Model-3 (adjusted model) revealed that older males and females from vulnerable population had 18% [AOR: 1.18; CI: 1.04,1.34] and 8% [AOR: 1.08; CI: 1.01,1.21] significantly higher odds of being physically frail in comparison to older males from non-vulnerable population respectively.

**Conclusions:**

Adverse socioeconomic circumstances such as low education, lower wealth and caste status that are associated with increased prevalence of physical frailty raise urgent questions both for public health practitioners and clinicians. The current findings may help to adapt public policies focusing on screening physical frailty in the clinical settings, especially among vulnerable populations as a marker of a possibly reversible vulnerability to adverse outcomes in old age.

**Supplementary Information:**

The online version contains supplementary material available at 10.1186/s12877-022-02891-1.

## Background

The rapid growth in proportion of older population has affected the healthcare settings worldwide and increased the need for developing long-term care services. Aging is often associated with accumulation of chronic conditions which in turn lead to disability among older adults [1–3]. Similarly, poor socioeconomic status may affect the individuals’ disease diagnosis, treatment adherence, resilience and physiological reserves [4–6].

Physical frailty phenotype among community-dwelling older adults, developed by Fried and colleagues refers to a clinical syndrome in which three or more of the following are present; exhaustion, weak grip strength, slow walk, low physical activity and unintentional weight loss [7, 8]. On the other hand, unlike physical frailty, vulnerability refers to social and environmental components affecting individuals’ wellbeing; including economic circumstances which themselves are recognized as determinants of health. However, physically frail individuals are vulnerable and environmental factors are more likely to increase their level of frailty and to have an independent effect on their level of vulnerability [9], suggesting a bidirectional linkage. Meanwhile, the influence of factors such as socioeconomic status in older age on increasing the risk of physical frailty is much debated among geriatric researchers [10].

Some of the earlier studies on physical frailty have demonstrated the social and environmental determinants of frailty including income, social participation and occupation [10, 11]. Studies showed that a holistic social vulnerability index in older adults including social factors from various domains of social support, social engagement and socioeconomic status (SES) predicts both cognitive decline and mortality [12, 13]. The increased mortality risk due to social vulnerability has also been observed among fittest older adults in a community setting reporting 0–1 out of 31 self-reported health deficits [14]. A couple of studies found that increasing social vulnerability, as measured by an index was associated with increased mortality and decreased survival rate in older adults [13–15], and other populations [16]. On the other hand, a study in China found physical frailty as a stronger predictor of mortality among higher socioeconomic groups than those with low SES [17].

Researchers have suggested that considering several indicators of SES such as income and education, the importance of which may diminish at older ages, some factors such as household wealth status, personal assets and social statuses remain important in assessing the status of older people [18–20]. Previous findings have also contributed to discussion of how to best measure SES in older age, where income, education and occupational status, may have more limited importance due to several old age pension schemes, changes in educational norms and retirement [21]. Besides, studies in India suggest that several socio-cultural and contextual factors such as social class and caste categories may also affect the health and wellbeing of older population, especially women [22–24]. Thus, we have broadened our consideration of social and economic vulnerability factors in the current study to create a more holistic representation of socioeconomic circumstances in Indian context by including caste status of individuals along with education and wealth status.

The need for an early care of physical frailty has been recently emphasised since it has an impact on patients, caregivers, healthcare professionals, and on society as a whole [25]. Although research on genetic and biological aspects of frailty is burgeoning, studies on the possible associations between factors of socioeconomic vulnerability and frailty are scarce. This is particularly important in India, as the population is rapidly aging with greater illiteracy and socioeconomic disadvantages. Thus, the objective of this study was to examine the relationship between socioeconomic vulnerability and frailty in community-dwelling older individuals in India. The study hypothesised that older adults who are socioeconomically vulnerable are more likely to be physically frail in India.

## Materials and methods

### Data

The data for this study came from the Longitudinal Aging Study in India (LASI), which was conducted in 2017–18. The Harvard T.H. Chan School of Public Health, the International Institute for Population Sciences (IIPS), and the University of Southern California (USC) collaborated on the study. The nationally representative longitudinal survey will collect crucial information on the physical, social, and cognitive well-being of India’s older citizens over a 25-year period. The LASI collected data of over 72,000 people aged 45 and over, as well as their spouses (of any age), across India’s states and union territories. The data collected were related to demographics, household economic status, chronic health conditions, symptom-based health conditions, functional health, mental health (cognition and depression), biomarkers, health insurance and healthcare utilization, family and social networks, welfare programmes, work and employment, retirement, satisfaction, and life expectations. The sample is based on a multistage stratified cluster sample design that includes three and four separate phases of rural and urban region selection. The survey gives scientific insights and allows for a standardised methodology that can be compared to other similar worldwide research. The LASI report contains information on sample design, survey instruments, fieldwork, data collecting and processing, and response rates [26]. For this investigation, the effective sample size after dropping the missing cases for physical frailty, was 14,652 older males and 15,899 older females aged 60 and over. The present study only analysed the data for older people defined as those who aged 60 years and above and hence the total sample size for the study was 30,551. The study was approved by an ethical committee of the Indian Council of Medical Research (ICMR) and was conducted in accordance with the relevant guidelines and regulations**.**

### Variable description

#### Outcome variable

##### Frailty

The physical frail older adults was assessed using an adapted version of the frailty phenotype described by Fried and colleagues**.** The physical frailty phenotype consists of five components: (1) self-reported exhaustion, (2) unintentional weight loss, (3) weak grip strength, (4) self-reported low physical activity, and (5) slow walking time. (1) Exhaustion was assessed using two questions from the Center for Epidemiologic Studies Depression (CES-D) scale: in the past week, how often do you feel “everything you did was an effort,” and “feel tired or low in energy” answered with “three or more days = 1” and “less than three days = 0”. (2) Unintentional weight loss was assessed using the question: “Do you think that you have lost weight in the last 12 months because there was not enough food at your household?” with responses “Yes = 1” and “No = 0.” (3) LASI measured handgrip strength in kilograms using a handheld Smedley’s Hand Dynamometer. The final handgrip strength score (in kg) was calculated as the average score (in kg) of two successive trials in the dominant hand, and was adjusted for the gender and body mass index, as shown in Table 1. (4) In LASI, respondents were asked about their physical activity: “How often do you take part in sports or vigorous activities, such as running or jogging, swimming, going to a health center or gym, cycling, or digging with a spade or shovel, heavy lifting, chopping, farm work, fast bicycling, cycling with loads: everyday, more than once a week, once a week, one to three times a month, or hardly ever or never?” The low physical activity was defined as: “One to three times a month or hardly ever or never = 1” and “once a week or more than once a week= 0”. LASI asked respondents to walk 4-m twice, and slowness was assessed by averaging the time (in seconds) taken in completing the 4 m (stratified by gender and height), as shown in Table 1. The overall physical frailty phenotype score lies between 0 and 5. Respondents with a score of 0 were classified as “not frail,” 1–2 as “pre-frail,” and three or higher as “frail.” The outcome variable was coded as binary i.e., physically frail which include (frail and pre-frail) and not frail. Assessment of the individual domains is described in Table 1.

**Table 1:** Criteria used to define physical frailty in LASI, 2017–18

Exhaustion	During the past week, how often did you feel: (a) tired or low in energy; (b) that everything you did was an effort. Less than three days = 0; More than three days =1.	
**Grip strength**	Average grip strength score in dominant hand (2 trials) using Smedley’s handheld dynamometer (adjusted for gender and body mass index (BMI))	
	**BMI**	**Cut-off for Grip strength**
	**For men**	
	BMI ≤ 24	≤ 29 kg
	BMI 24.1–26	≤ 30 kg
	BMI 26.1–28	≤ 30 kg
	BMI > 28	≤ 32 kg
	**For women**	
	BMI ≤ 23	≤ 17 kg
	BMI 23.1–26	≤ 17.3 kg
	BMI 26.1–29	≤ 18 kg
	BMI > 29	≤ 21 kg
**Walk time**	In the LASI, respondents were asked to walk 4 m twice. The time taken to walk was recorded in seconds each time, and the average time taken in both trials was calculated. (stratified by gender and height (gender-specific cut-off a medium height)	
		Cut-off for Time to Walk 4-m
	**Men**	
	Height ≤ 173 cm	≥ 7 s
	Height > 173 cm	≥ 6 s
	**Women**	
	Height ≤ 159 cm	≥ 7 s
	Height > 159 cm	≥ 6 s
**Weight loss**	Do you think that you have lost weight in the last 12 months because there was not enough food at your household?” Yes = 1, No = 0.	
**Physical activity**	How often do you take part in sports or vigorous activities, such as running or jogging, swimming, going to a health center or gym, cycling, or digging with a spade or shovel, heavy lifting, chopping, farm work, fast bicycling, cycling with loads? One to three times a month or hardly ever or never = 1, once a week or more than once a week = 0	
**Total score**	5 Items: 0 deficits = non-frail, 1–2 deficits = pre-frail, 3+ deficits = frail, allowed missing 1–2 items, missing imputed with 0 (sum of available items)	

### Explanatory variables

#### Main explanatory variable

##### Vulnerability measures

To understand multiple vulnerabilities, a variable integrating the three dimensions of vulnerability based on education, wealth, and caste was constructed as they were used in the two Human Poverty Indexes and the Multidimensional Poverty Index (instead of the caste they used health). For constructing the individual index, low education is classified as those older adults who did not complete 5 years of schooling. For education, an older adult is considered to be deprived or vulnerable if he/she reported in his/her individual survey that he/she had not completed 5 years of schooling. This cut off is chosen because people with only a few years of education have been found to have health-seeking behaviour similar to those with no education [27]. Using household consumption data, the monthly per-capita consumption expenditure (MPCE) quintile was determined. The sample households were canvassed using sets of 11 and 29 questions on food and non-food expenses, respectively. Food spending was gathered over a seven-day reference period, whereas non-food expenditure was collected over 30-day and 365-day reference periods. The 30-day reference period has been used to standardise food and non-food expenses. The MPCE is calculated and used to summarise consumption. The variable was divided into five quintiles i.e., from poorest to richest. Those who belonged to the poorest or poorer wealth quintile have been considered as economically “poor” and middle, richer, and richest are “non-poor”. For caste, an older adult is considered vulnerable if he or she belonged to the Scheduled Caste or Scheduled Tribe.

In this way, using the three dimensions of vulnerability based on education, wealth, and caste, eight categories of vulnerability were possible: education, wealth and caste; education and wealth; education and caste; wealth and caste; education only; wealth only; caste only and anyone. The first four categories classified vulnerability in multiple dimensions, the next three in one dimension and the last category in any-one [27].

#### Other explanatory variables

Age was recoded as young-old (60–69 years), old-old (70–79 years) and oldest-old (80+ years); living arrangement was recoded as living alone, living with spouse, living with children and living with others; marital status was recoded as currently married, widowed and others (others included divorced/ separated/ never married) [28]; work status was recoded as never worked, currently working, currently not working and retired [29]. Further, social participation was recoded as no and yes. Respondents were said to be participating in social activities if they participate in any of the following; eat out of house (Restaurant/Hotel); go to park/ beach for relaxing/ entertainment; play cards or indoor games; play out door games/ sports/ exercise/ jog/ yoga; visit relatives /friends; attend cultural performances /shows/ Cinema; attend religious functions /events such as *bhajan*/ *satsang*/ prayer; attend political/ community/ organization group meetings; read books/ newspapers/ magazines; watch television/ listen to radio and use a computer for e-mail/net surfing etc. [26].

Self-rated health was recoded as (good which includes excellent, very good, and good whereas poor includes fair and poor) [30]. The probable major depression with symptoms of dysphoria was calculated using the CIDI-SF (Short Form Composite International Diagnostic Interview). This scale estimates a probable psychiatric diagnosis of major depression and has been validated in field settings and widely used in population-based health surveys [26]. The scale was validated for older adults [31]. The respondent was considered to be depressed if the score was 3 and above in the scale of 0–10 [26, 32]. Difficulty in activities of daily living (ADL) was recoded as (no and yes). The ability or inability to perform ADLs is used to assess a person’s functional state, particularly for persons with impairments and those who are older [33]. If the respondent reported any difficulty in ADLs then difficulty in ADL was coded as yes otherwise no. Difficulty in instrumental ADL (IADL) was recoded as (no and yes). These tasks are necessary for independent functioning in the community. If the respondent reported any difficulty in IADLs then difficulty in IADL was coded as yes and otherwise no [34]. Morbidity status was categorized as 0 “no morbidity”, 1 “any one morbid condition” and 2+ “co-morbidity” [35]. Religion was recoded as Hindu, Muslim, Christian, and Others. Place of residence was recoded as (rural and urban). The regions were recoded as North, Central, East, Northeast, West, and South.

### Statistical analysis

The study carried out bivariate analysis to observe the association between dependent and independent variables. Further, multivariable binary logistic regression analysis [36] was used to fulfil the objective of the study. The results were presented in the form of odds ratio (OR) with a 95% confidence interval (CI).

Model-1 provides the unadjusted estimates whereas model-2 and 3 provide the adjusted estimates (adjusted for age, living arrangement, marital status, working status, social participation, self-rated health, depression, difficulty in ADL, difficulty in IADL, morbidity status, religion and place of residence and regions of India). The unadjusted and adjusted estimates of interaction of sex and vulnerability on physical frailty are provided in model-1 and model-3, respectively [34, 37]. While analysing the interaction effects, a new categorical variable is created based on vulnerability and sex.

## Results

Table 2 presents the socio-economic profile of the study participants. About 2.5% of older men and 8.6% older women were living alone. A proportion of 16.5% of older men and 53.5% of older women were widowed. About 43.1% of older men and 19.2% older women were currently working. Also, 7.4 and 9.7% of older men and women respectively had no social participation. A proportion of 46.7 and 50.2% of older men and women had poor self-rated health respectively. Nearly 7.5% of older men and 9.7% of older women were suffering from depression. Similarly, 20.1 and 38.0% of older men and 25.4 and 56.2% of older women had difficulty in ADL and IADL respectively. And, 22.1 and 25.3% of older men and women had two or more morbidities.

**Table 2:** Socio-economic profile of the study participants, 2017–18

Background characteristics	Male		Female	
	Sample	%	Sample	%
**Age**				
Young-old	8632	58.9	9524	59.9
Old-old	4453	30.4	4672	29.4
Oldest-old	1567	10.7	1703	10.7
**Living arrangement**				
Living alone	362	2.5	1369	8.6
Living with spouse	3623	24.7	2435	15.3
Living with children	10,125	69.1	11,004	69.2
Living with others	541	3.7	1091	6.9
**Marital status**				
Currently married	11,887	81.1	7091	44.6
widowed	2414	16.5	8507	53.5
Others	350	2.4	301	1.9
**Working status**				
Never worked	551	3.8	7415	46.6
Currently working	6308	43.1	3059	19.2
Currently not working	5818	39.7	5129	32.3
Retired	1973	13.5	295	1.9
**Social participation**				
No	1080	7.4	1542	9.7
Yes	13,572	92.6	14,357	90.3
**Self-rated health**				
Good	7806	53.3	7918	49.8
Poor	6846	46.7	7981	50.2
**Depression**				
No	13,559	92.5	14,358	90.3
Yes	1093	7.5	1541	9.7
**Difficulty in ADL**				
No	11,714	80.0	11,862	74.6
Yes	2938	20.1	4037	25.4
**Difficulty in IADL**				
No	9090	62.0	6958	43.8
Yes	5562	38.0	8941	56.2
**Morbidity status**				
0	7261	49.6	7101	44.7
1	4154	28.4	4778	30.1
2+	3237	22.1	4020	25.3
**Religion**				
Hindu	12,126	82.8	13,138	82.6
Muslim	1599	10.9	1679	10.6
Christian	381	2.6	503	3.2
Others	545	3.7	580	3.7
**Place of residence**				
Rural	10,664	72.8	11,028	69.4
Urban	3988	27.2	4871	30.6
**Region**				
North	1842	12.6	2064	13.0
Central	3322	22.7	3078	19.4
East	3631	24.8	3642	22.9
Northeast	431	2.9	479	3.0
West	2391	16.3	2861	18.0
South	3036	20.7	3775	23.7
**Total**	14,652	100	15,899	100

Table 3 presents the percentage of older men and women by various vulnerabilities and their combinations. It was found that 10.5 and 14.5% of older men and women were in the overall vulnerable category respectively. Additionally, 73.1 and 87.3% of older men and women were from any one vulnerable category respectively.

**Table 3:** Percentage of older males and females by various vulnerabilities in India, 2017–18

Variables	Male		Female		***p***-value
	Sample	%	Sample	%	
**Wealth**					
Not vulnerable	8734	57.9	9074	55.4	< 0.001
Vulnerable	6364	42.2	7292	44.6	< 0.001
**Caste**					
Not vulnerable	11,097	73.5	11,865	72.5	< 0.001
Vulnerable	4001	26.5	4501	27.5	< 0.001
**Educational status**					
Not vulnerable	7079	46.9	3052	18.7	0.002
Vulnerable	8019	53.1	13,314	81.4	< 0.001
**Wealth and education**					
Not vulnerable	11,090	73.5	9886	60.4	< 0.001
Vulnerable	4008	26.6	6480	39.6	0.003
**Caste and education**					
Not vulnerable	12,310	81.5	12,127	74.1	< 0.001
Vulnerable	2788	18.5	4239	25.9	< 0.001
**Caste and wealth**					
Not vulnerable	12,950	85.8	13,899	84.9	< 0.001
Vulnerable	2148	14.2	2467	15.1	< 0.001
**Any one**					
Not vulnerable	4068	27.0	2077	12.7	0.013
Vulnerable	11,030	73.1	14,289	87.3	< 0.001
**Overall vulnerability**					
Not vulnerable	13,509	89.5	13,999	85.5	< 0.001
Vulnerable	1589	10.5	2367	14.5	0.042

Table 4 presents the percentage distribution of physical frailty among older men and women by their background characteristics. It was revealed that the prevalence of physical frailty was high among older men with vulnerability status (31.4% vs 26.9%); however, in case of older women, the prevalence was slightly high in those with vulnerability status (32.7% vs 32.1%). Additionally, it was found that there were significant gender differentials (differences: 4.8%; *p* < 0.001) in physical frailty among older adults. The prevalence of physical frailty was higher among older men and women in the higher age group, those with no social participation, with poor health outcomes such as depression, poor self-rated health and difficulty in functional health and those residing in rural areas of the country. Higher percentage of older male and female from Muslim religion were physically frail. The prevalence of frailty was high among older male and females from rural areas. Also, the older adults from eastern part of India had higher prevalence of frailty.

**Table 4:** Percentage distribution of physical frailty among older males and females by their background characteristics in India, 2017–18

Background characteristics	Male	Female	Differences	***p***-value
**Vulnerability status**				
No	26.9	32.1	5.2	< 0.001
Yes	31.4	32.7	1.4	0.043
**Age**				
Young-old	19.2	23.4	4.2	< 0.001
Old-old	34.8	41.0	6.2	< 0.001
Oldest-old	51.2	56.5	5.4	< 0.001
**Living arrangement**				
Living alone	46.9	38.9	−8.0	0.946
Living with spouse	27.0	27.7	0.7	0.183
Living with children	26.3	31.2	4.9	< 0.001
Living with others	36.5	42.8	6.4	0.018
**Marital status**				
Currently married	24.8	26.1	1.3	0.059
widowed	39.2	37.6	−1.6	0.827
Others	34.4	21.7	−12.7	0.116
**Working status**				
Never worked	36.1	32.4	−3.7	0.339
Currently working	14.0	16.1	2.1	< 0.001
Currently not working	42.4	41.6	−0.8	0.248
Retired	23.5	29.9	6.4	0.070
**Social participation**				
No	37.6	44.3	6.7	0.034
Yes	26.6	30.9	4.3	< 0.001
**Self-rated health**				
Good	20.4	24.4	4.0	< 0.001
Poor	35.3	39.8	4.5	< 0.001
**Depression**				
No	26.0	30.7	4.7	< 0.001
Yes	43.8	45.4	1.6	0.012
**Difficulty in ADL**				
No	23.7	27.3	3.6	< 0.001
Yes	42.1	46.5	4.4	0.007
**Difficulty in IADL**				
No	20.4	24.1	3.7	< 0.001
Yes	38.7	38.4	−0.3	0.472
**Morbidity status**				
0	24.5	29.3	4.8	< 0.001
1	28.1	32.8	4.7	< 0.001
2+	33.0	36.5	3.5	< 0.001
**Religion**				
Hindu	27.1	31.8	4.7	< 0.001
Muslim	29.8	37.2	7.4	< 0.001
Christian	25.3	32.2	6.8	0.015
Others	26.9	25.2	−1.7	0.472
**Place of residence**				
Rural	28.0	33.8	5.8	< 0.001
Urban	25.8	28.4	2.7	< 0.001
**Region**				
North	24.2	29.2	5.0	< 0.001
Central	28.9	35.7	6.8	< 0.001
East	31.3	36.9	5.6	< 0.001
Northeast	19.5	26.1	6.6	0.003
West	19.5	26.1	6.6	< 0.001
South	30.2	31.7	1.5	< 0.001
**Total**	27.4	32.2	4.8	< 0.001

*p*-value based in proportion test; difference = Female - Male

**Fig. 1:**
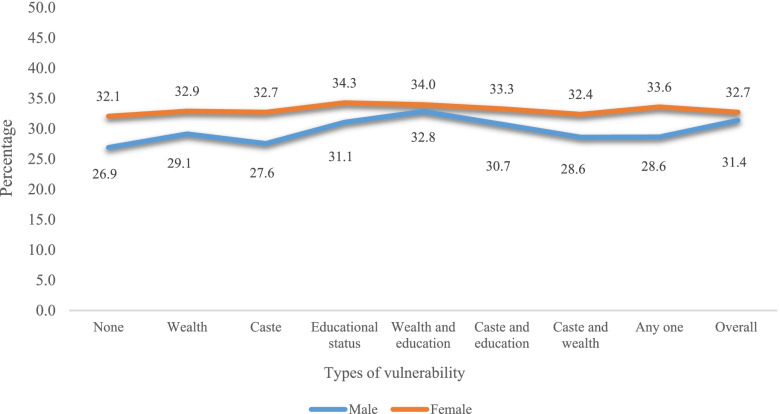
Percentage prevalence of physical frailty among older males and females by various vulnerabilities

Figure 1 presents the percentage of physical frailty among older males and females with respective vulnerability status. It was found that vulnerable older females had high prevalence of frailty in all categories in reference to older males. Table S1 presents the percentage prevalence of each components of physical frailty by various vulnerabilities stratified by sex (supplementary file).

Table 5 presents the logistic regression estimates of physical frailty among older adults by their background characteristics. Model-1 revealed that the vulnerable older adults had 8% significantly higher odds of being frail in comparison to non-vulnerable older adults [UOR: 1.08; CI: 1.01, 1.16]. Moreover, the same model revealed that older females had 32% significantly higher odds of being frail in reference to older males [UOR: 1.32; CI: 1.26,1.39]. It was further found that the odds of frailty were significantly high among older females from vulnerable category in comparison to older males from non-vulnerable category [UOR: 1.34; CI: 1.27,1.46].

**Table 5:** Logistic regression estimates for physical frailty among older adults by their vulnerability status in India, 2017–18

Background characteristics	Model-1	Model-2	Model-3
	UOR	AOR	AOR
**Vulnerability status**			
No	Ref.	Ref.	
Yes	1.08*(1.01,1.16)	1.14*(1.06,1.24)	
**Sex**			
Male	Ref.	Ref.	
Female	1.32*(1.26,1.39)	0.97 (0.9,1.03)	
**Sex # Vulnerability status**			
Male # No	Ref.		Ref.
Female # No	1.34*(1.27,1.42)		0.97 (0.90.1.04)
Male # Yes	1.16*(1.03,1.29)		1.18*(1.04,1.34)
Female # Yes	1.33*(1.21,1.46)		1.08*(1.01,1.21)

*if *p* < 0.05; *AOR* Adjusted odds ratio, *UOR*: Unadjusted odds ratio; #: interaction effect; Model-1 is the unadjusted model; Model-2 and 3 are the adjusted models adjusted for the background characteristics (age, living arrangement, marital status, working status, social participation, self-rated health, depression, difficulty in ADL, difficulty in IADL, morbidity status, religion, place of residence, region)

Model-2 which represents the adjusted estimates revealed that older adults from vulnerable category had 14% significantly higher odds of being physically frail in reference to non-vulnerable category [AOR: 1.14; CI: 1.06,1.24]. The adjusted model further revealed that there were no significant gender differentials in physical frailty among older adults. Model-3 (adjusted model) revealed that older males and females from vulnerable category had 18% [AOR: 1.18; CI: 1.04,1.34] and 8% [AOR: 1.08; CI: 1.01,1.21] significantly higher odds of being physically frail in comparison to older males from non-vulnerable category respectively.

## Discussion

This population-based cross-sectional study depicted the vulnerability factors that are associated with frailty in older adults. The higher prevalence of physical frailty, with 27.4% older men and 32.2% older women being physically frail suggests that frailty in community-dwelling older Indian adults is common and needs to be prioritized in policy formation for geriatric population. This is consistent with previous studies that concluded that irrespective of definitions of frailty, the prevalence is higher in India compared to other low and middle income countries [38, 39]. In this regard, future studies on urban-rural and regional variations of old age frailty in India are warranted. Although increasing age is a risk factor for physical frailty, not all the older people are frail which suggests for further investigation on reasons other than normal aging process to augment this condition in aged population. We found that among older Indian adults, those with greater vulnerability were more likely to be frail in comparison to those with no social vulnerability. The risk for being frail was especially higher among older women who are more vulnerable than older men with same vulnerability status.

The most evident reason for older adults who are socioeconomically advantaged turning to be less frail than those who are disadvantaged is their potential for prevention suggested by a spontaneous reversibility of frailty in individuals in their early stages of frailty. Factors such as availability of resources, increased access to healthcare and health literacy among high economic groups in India which help older adults detect their disability and diseases earlier and prevent from further accumulation of such conditions are supportive of this argument [40, 41]. However, this potential in wealthy and higher social groups is challenged by the lack of specific public interventions of proven effectiveness and efficacy in socioeconomically poor frail individuals [42]. This may be substantiated by studies on the association of lack of social resources among the poor people with their functional decline in old age [43]. The previous findings in India suggesting that rural populations are disproportionately affected by poor health outcomes due to barriers to diagnosis also suggest their increased vulnerability to physical frailty in old age [44].

The female disadvantage in physical frailty prevalence observed in our study is consistent with other research in developing countries. Several cross-sectional and longitudinal studies in China found that the prevalence and incidence of physical frailty was significantly higher in women [45, 46]. In comparison to China, treatment levels of various chronic diseases were lower in India and women in particular [47], which might also have worsened the physical frailty status of vulnerable older Indian female population. Similarly, studies in low- and middle income countries observed that differential exposure and differential vulnerability to social conditions and biological factors in men and women are associated with gender differences in physical functioning and mental health status in older ages [48–51]. However, the interaction of sex and vulnerability on physical frailty revealed that older men who are socioeconomically vulnerable are more likely to be physically frail than women who are vulnerable after adjusting for several confounders. This suggests that several health-related variables might have led to increased physical frailty in women, whereas; a poor SES results in higher risk of physical frailty in men.

Studies in various settings have revealed a marked educational gradient in frailty, where older individuals with no formal education or little education showed greater odds of physical frailty than their counterparts with higher education [49, 52, 53]. Several studies in India also found lower levels of disability and physical frailty among older adults with higher levels of education and wealth [38, 54–58]. This low education- frailty association consistently found in the current study, can be attributed to the increased material, health and behavioural resources available to those who are highly educated [49, 59, 60].

With regard to the prevalence of physical frailty among different ethnic groups, the findings are inconclusive. Some studies among Mexican Americans and European Americans showed higher prevalence in ethnic minorities [61, 62], whereas several studies have shown that African-American race in comparison to white races was independently associated with frailty [63, 64]. This suggests that higher prevalence of frailty among vulnerable ethnic groups in India (Scheduled Castes and Scheduled Tribes) requires future investigation. Furthermore, frailty prevalence differs between the regions, mainly because of great regional disparities in health care, which are related to socioeconomic characteristics [65]. This is substantiated by findings of other studies showing that rural residents in India who have low income and educational levels and less access to health services and insurance, which lead to poorer health are at increased risk of becoming physically frail [38].

This study has several limitations that should be addressed in the studies in future. First, our study was cross-sectional in design, and thus the direction of causality could not be ascertained. It is possible that frailty in old age may accelerate the vulnerability, for example, frail older adults being economically poor. It is also possible that frail older adults are less likely to participate in social activity, which is associated which in turn increase their vulnerability to frailty, suggesting the other pathways which are not considered in the current study. These mechanisms therefore warrant further investigation to determine what other factors may account for the unexplained variance between vulnerability factors and physical frailty in older individuals. Future studies with follow-up rounds of LASI survey also should be conducted to better understand the aspect by exploring other vulnerability factors.

## Conclusion

The findings highlight the higher prevalence of physical frailty among community-dwelling older adults and among those who had lower educational, wealth or caste status or a combination of these factors. Adverse socioeconomic circumstances that are associated with increased prevalence of physical frailty raise urgent questions both for public health practitioners and clinicians. The current findings may help to adapt public policies focusing on screening physical frailty in the clinical settings, especially among vulnerable populations as a marker of a possibly reversible vulnerability to adverse outcomes in old age. Also, the observed relationships between several socioeconomic factors as determinants of adverse late-life physical frailty need further research.

## Supplementary Information

**Additional file 1.**
