# Supplementary material for: Socioeconomic vulnerability and frailty among community-dwelling older adults: cross-sectional findings from longitudinal aging study in India, 2017–18
Source: BMC Geriatr. 2022 Mar 14;22:201. doi: 10.1186/s12877-022-02891-1 (PMC8919576; doi:10.1186/s12877-022-02891-1)
Supplement: Supplementary file 1 — Additional file 1. [file 12877_2022_2891_MOESM1_ESM.docx]

| **Table-S1.** Percentage prevalence of physical frailty components by various vulnerabilities stratified by sex in India, 2017-18 | | | | | | | | | | |
| --- | --- | --- | --- | --- | --- | --- | --- | --- | --- | --- |
| **Variables** | **Exhaustion** | | **Grip strength** | | **Walk time** | | **Weight loss** | | **Physical activity** | |
|  | **Male** | **Female** | **Male** | **Female** | **Male** | **Female** | **Male** | **Female** | **Male** | **Female** |
| **Wealth** |  |  |  |  |  |  |  |  |  |  |
| Not vulnerable | 35.4 | 37.9 | 64.8 | 53.4 | 12.9 | 22.9 | 5.1 | 5.0 | 63.1 | 80.9 |
| Vulnerable | 37.3 | 38.5 | 69.6 | 54.4 | 14.0 | 22.5 | 6.7 | 6.1 | 64.2 | 82.2 |
| **Caste** |  |  |  |  |  |  |  |  |  |  |
| Not vulnerable | 36.2 | 37.7 | 65.7 | 53.4 | 13.0 | 23.0 | 5.0 | 4.8 | 64.4 | 82.8 |
| Vulnerable | 36.1 | 39.3 | 70.0 | 54.9 | 14.3 | 22.0 | 8.0 | 7.3 | 61.3 | 77.9 |
| **Educational status** |  |  |  |  |  |  |  |  |  |  |
| Not vulnerable | 34.0 | 32.7 | 61.3 | 47.1 | 10.7 | 17.9 | 3.4 | 1.9 | 64.3 | 83.0 |
| Vulnerable | 38.1 | 39.4 | 71.7 | 55.4 | 15.8 | 23.8 | 7.8 | 6.3 | 63.0 | 81.1 |
| **Wealth and education** |  |  |  |  |  |  |  |  |  |  |
| Not vulnerable | 35.4 | 37.6 | 64.6 | 52.9 | 12.6 | 22.7 | 4.8 | 4.8 | 63.4 | 81.2 |
| Vulnerable | 38.2 | 38.9 | 73.1 | 55.3 | 15.6 | 22.8 | 8.4 | 6.6 | 64.3 | 82.0 |
| **Caste and education** |  |  |  |  |  |  |  |  |  |  |
| Not vulnerable | 35.6 | 37.6 | 65.8 | 53.2 | 12.7 | 22.8 | 5.0 | 4.8 | 64.2 | 82.6 |
| Vulnerable | 38.7 | 39.6 | 71.4 | 55.8 | 16.2 | 22.6 | 9.3 | 7.5 | 61.0 | 78.3 |
| **Caste and wealth** |  |  |  |  |  |  |  |  |  |  |
| Not vulnerable | 36.1 | 37.9 | 65.8 | 53.9 | 13.1 | 22.9 | 5.4 | 5.1 | 64.1 | 82.1 |
| Vulnerable | 36.3 | 39.4 | 72.9 | 53.8 | 14.7 | 21.6 | 8.2 | 7.7 | 60.7 | 78.0 |
| **Any one** |  |  |  |  |  |  |  |  |  |  |
| Not vulnerable | 33.8 | 31.7 | 59.5 | 47.9 | 10.5 | 17.0 | 2.9 | 1.5 | 64.5 | 83.2 |
| Vulnerable | 37.1 | 39.1 | 69.5 | 54.7 | 14.4 | 23.5 | 6.8 | 6.1 | 63.3 | 81.2 |
| **Vulnerability status** |  |  |  |  |  |  |  |  |  |  |
| Not vulnerable | 35.9 | 37.9 | 66.0 | 53.8 | 13.0 | 22.8 | 5.3 | 5.1 | 64.0 | 82.0 |
| Vulnerable | 38.2 | 39.5 | 73.8 | 54.1 | 16.4 | 22.0 | 9.6 | 7.7 | 60.6 | 78.3 |
| **Total** | 36.2 | 38.1 | 66.8 | 53.9 | 13.4 | 22.7 | 5.8 | 5.5 | 63.6 | 81.5 |
